# Supplementary material for: Evaluation of Baseline Characteristics and Prognostic Factors in Multisystemic Inflammatory Syndrome in Children: Is It Possible to Foresee the Prognosis in the First Step?
Source: J Clin Med. 2022 Aug 8;11(15):4615. doi: 10.3390/jcm11154615 (PMC9369528; doi:10.3390/jcm11154615)
Supplement: Supplementary file 1 [file jcm-11-04615-s001.zip › jcm-1790439-supplementary.pdf]

Supplementary Materials

Table S1: Treatments of the MIS-C patients

	All MIS-C patients (n=99)
Primary treatment regime, *n (%)*	
IVIG plus steroid	85 (85.9)
IVIG only	9 (9.1)
Steroid only	3 (3.0)
IVIG or steroid not used	2 (2.0)
IVIG dosage, *n (%)*	
1 gr/kg totally	16 (16.2)
2 gr/kg totally	78 (78.8)
Not used	5 (5.1)
Steroid dosage, *n (%)*	
1 – 2 mg/kg/day	78 (78.8)
10 – 20 mg/kg/day	5 (5.1)
1 gr/day	5 (5.1)
Not used	11 (11.1)
Antithrombotic therapy, *n (%)*	
LMWH plus aspirin	52 (52.5)
Aspirin only	31 (31.3)
LMWH only	8 (8.1)
Not used	8 (8.1)
Vasoactive and inotropic drug usage, *n (%)*	15 (15.2)
Biologic agent usage, *n (%)*	
IL-1 inhibitor (anakinra)	6 (6.1)
IL-6 inhibitor (tocilizumab)	1 (1.0)
Not used	92 (92.9)
Respiratory support, *n (%)*	
Oxygen only (normal or high-flow)	11 (11.1)
Mechanical ventilation	4 (4.0)
Not indicated	84 (84.8)
Plasmapheresis, *n (%)*	3 (3.0)

Abbreviation: IVIG, intravenous immunoglobulin; LMWH, low molecular weight heparin.
